# Feasibility Randomised Trial Comparing Two Forms of Mental Health Supported Accommodation (Supported Housing and Floating Outreach); a Component of the QuEST (Quality and Effectiveness of Supported Tenancies) Study

**DOI:** 10.3389/fpsyt.2019.00258

**Published:** 2019-04-17

**Authors:** Helen Killaspy, Stefan Priebe, Peter McPherson, Zohra Zenasni, Paul McCrone, Sarah Dowling, Isobel Harrison, Joanna Krotofil, Christian Dalton-Locke, Rose McGranahan, Maurice Arbuthnott, Sarah Curtis, Gerard Leavey, Rob MacPherson, Sandra Eldridge, Michael King

**Affiliations:** ^1^Division of Psychiatry, University College London, London, United Kingdom; ^2^Camden and Islington NHS Foundation Trust, London, United Kingdom; ^3^Unit for Social and Community Psychiatry, Queen Mary University of London, London, United Kingdom; ^4^Pragmatic Clinical Trials Unit, Queen Mary University of London, London, United Kingdom; ^5^King’s Health Economics, King’s College London, London, United Kingdom; ^6^North London Service User Research Forum, Division of Psychiatry, University College London, London, United Kingdom; ^7^Department of Geography, Durham University, Durham, United Kingdom; ^8^Bamford Centre for Mental Health and Wellbeing, University of Ulster, Londonderry, United Kingdom; ^9^2Gether NHS Foundation Trust, Gloucester, United Kingdom

**Keywords:** QuEST, mental health, supported accommodation, feasibility, trial, supported housing, floating outreach

## Abstract

**Background:** Mental health supported accommodation services are implemented across England, usually organised into a ‘step-down’ care pathway that requires the individual to repeatedly move as they gain skills and confidence for more independent living. There have been no trials comparing the effectiveness of different types of supported accommodation, but two widely used models (supported housing and floating outreach) have been found to provide similar support. We aimed to assess the feasibility of conducting a large-scale trial comparing these two models.

**Methods:** Individually randomised, parallel group feasibility trial in three regions of England (North London, East London, and Cheltenham and Gloucestershire). We aimed to recruit 60 participants in 15 months, referred to supported accommodation, randomly allocated on an equal basis to receive either a local supported housing or floating outreach service. We assessed referrals to the trial, participants recruited, attrition, time from recruitment to moving into either type of supported accommodation, and feasibility of masking. We conducted a process evaluation to examine our results further.

**Results:** We screened 1,432 potential participants, of whom 17 consented to participate, with 8 agreeing to randomisation (of whom 1 was lost to attrition) and 9 participating in naturalistic follow-up. Our process evaluation indicated that the main obstacle to recruitment was staff and service user preferences for certain types of supported accommodation or for specific services. Staff also felt that randomisation compromised their professional judgement.

**Conclusions:** Our results do not support investment in a large-scale trial in England at this time.

**Trial registration:** UK CRN Portfolio database, Trial ID: ISRCTN19689576.

**Trial funding:** National Institute of Health Research (RP-PG-0707-10093).

## Introduction

In England, one third of working-age adults with severe mental health problems (around 60,000 people) reside in supported accommodation provided by health and social services and housing associations (1, 2). These services have been categorised into three main types (3): residential care homes (offering high levels of longer-term support, 24 h a day to individuals with high needs), supported housing (offering time-limited, building-based support to individuals in single or shared tenancies), and floating outreach (offering flexible, visiting support to people in a permanent tenancy). The costs vary from around £150 per person per week for floating outreach to around £500 for residential care (4). The annual cost to the health and social care budget is therefore hundreds of millions of pounds.

The majority of people who require these services have complex mental health needs and functional impairments that impair their ability to manage activities of daily living. In England, individuals often graduate from supported housing services to floating outreach as their skills and confidence to manage their own tenancy increase. However, previous studies suggest that there are few differences in the amount and type of support provided to people in these two models and individuals dislike having to move home repeatedly as they progress along this pathway (3–5). Furthermore, varying preferences for different models have been found between service users, staff and family members, with service users tending to prefer more independent, permanent accommodation and staff and family members preferring the person to reside in more supported settings (6–8). Conversely, some service users report that independent tenancies are socially isolating (9). In addition, within a highly pressured mental health system, it is likely that allocation of accommodation may be driven more by availability than clinical need.

There have been very few trials comparing the effectiveness of different models of mental health supported accommodation and those that have been conducted have tended to focus on homeless populations and none have been conducted in the United Kingdom (10, 11). We therefore do not know whether individuals with severe and complex mental health needs are following the most cost-effective routes to independence, i.e. whether support delivered to service users in their own homes through floating outreach is more effective than the time-limited ‘step-down’ approach provided in staffed supported housing facilities. In short, we do not know whether more tailored support delivered to service users in their own homes through floating outreach is more acceptable, more individualised, and more cost-effective than a standard level of support provided in staffed facilities. There are similarities here with the ‘train and place’ and ‘place and train’ supported employment models, the latter being most commonly delivered through Individual Placement and Support, which has been shown to be more effective at helping service users to achieve competitive employment than graduated, sheltered employment schemes (12). The clinical uncertainty relating to supported accommodation justifies assessment through a randomised controlled trial, but, given the logistic challenges, there is first a need to assess the feasibility of conducting such a trial.

This study comprised the fourth component of the QuEST study (Quality and Effectiveness of Supported Tenancies for people with mental health problems; http://www.ucl.ac.uk/quest), the first national research programme to investigate the provision, quality, and effectiveness of mental health supported accommodation services in England. The programme included adaptation of a service quality assessment tool and client satisfaction tool for these settings (13, 14), a national survey (15), a cohort study investigating longer-term outcomes and a qualitative investigation of staff and service user experiences (15). This paper reports on the feasibility randomised trial comparing the effectiveness of supported housing and floating outreach.

We aimed to assess the feasibility, sample size, and most appropriate outcomes for a large-scale trial to compare the clinical and cost-effectiveness of these two models of mental health supported accommodation commonly used in England. Specifically, we aimed to establish whether participant recruitment and randomisation to different types of supported accommodation was possible, given the potentially complex logistics involved.

## Materials and methods

### Design

Individually randomised, parallel group feasibility trial.

### Protocol

The full trial protocol can be accessed via the corresponding author’s institution’s website (www.ucl.ac.uk/quest/protocol).

### Setting

The feasibility trial was conducted in three sites that provided both types of supported accommodation and where the study team had good links (North London – Camden and Islington; East London – Tower Hamlets, Newham, Hackney; Gloucestershire and Cheltenham).

### Sample Size

As this was a feasibility trial, a formal sample size calculation was not required but we set a target of recruiting and randomising 60 participants from across the three study sites over 15 months. We aimed to recruit 20 participants per site on the basis that any fewer would make a large-scale trial unfeasible.

### Inclusion/Exclusion Criteria

All service users in the three study sites referred to either supported housing or floating outreach services who had housing rights in the local area and were subject to the Care Programme Approach (to ensure input from a community mental health team for all participants) were eligible for inclusion. Those who lacked capacity to give informed consent were not eligible.

### Recruitment Process

Each of the three sites had a system for referral of service users to local supported accommodation services. All those referred to supported housing or floating outreach were considered for potential participation in the study. We first met with the relevant staff at each site to clarify the purposes of the study and local referral processes. A researcher at each study site liaised with the personnel coordinating the referrals system and clinicians making referrals. They identified appropriate referrals eligible for participation in the study who were then contacted for informed consent to participate. We did not contact individuals whom the clinical team considered inappropriate. We were aware of the potential recruitment challenges facing us and therefore, in addition, service users who did not consent to randomisation were offered participation in a naturalistic cohort whereby they gave informed consent to complete the same research interviews as trial participants but their supported accommodation was not allocated randomly. Recruitment took place over 15 months from June 2015.

After 6 months, we decided to adjust our approach to try to increase recruitment. In addition to the processes described above, researchers met with the managers of acute inpatient wards and community mental teams in the three sites to identify any individuals being considered for referral to supported housing who had not yet been referred.

### Randomisation and Masking

Participants were randomly allocated on an equal basis to receive either a local supported housing or floating outreach service. Computer-generated randomisation was carried out independently of the research team by a statistician from the Pragmatic Clinical Trials Unit at Queen Mary’s University London and stratified by site. The outcome of randomisation was communicated to the QuEST project manager who informed the local referrals coordinator and referrer, who then processed the participant’s supported accommodation allocation accordingly.

We explored the feasibility of using a telephone interview to collect follow-up data from service users. At 12-month follow-up, the researcher met with the service user participant and then telephoned a second researcher (masked to the participant’s supported accommodation allocation) who completed one instrument from the interview battery (Manchester Short Assessment of Quality of Life – MANSA (16)) with the participant. This measure was selected as all others would have invalidated the masking by revealing the participant’s allocation.

### Comparison Services

Supported housing services provided a constant level of staffing on-site to a number of service users living in individual or shared tenancies with the expectation of move-on within 2 years. Floating outreach services provided visiting support of flexible intensity to service users living in a permanent independent tenancy. In terms of the simple taxonomy for supported accommodation (STAX-SA) (17), supported housing services are Types 2 and 3, whilst floating outreach services are Type 4.

### Content of Care

Content of care provided in all services was assessed using the Quality Indicator for Rehabilitative Care – Supported Accommodation version (QuIRC-SA) (13), completed with the service manager once for each service, 6 months after the participant was randomised (assuming they had moved to the allocated accommodation by then). This comprehensive, standardised measure provides descriptive data on resources and ratings of the service’s quality of care on seven domains and was completed once per service.

### Data Collection

We collected the following metrics to inform the feasibility of a larger trial: number of referrals to the trial; number of participants recruited; attrition (i.e. number of participants who withdrew consent to continue with the research, declined to move to the allocated service, or could not be located at follow-up); and time from recruitment to moving into either type of supported accommodation. We assessed the feasibility of using a range of potential standardised outcome measures [Client Assessment of Treatment – Supported Accommodation version (14), Clinician Alcohol and Drugs Scale (18), The Life Skills Profile (19), MANSA (16), Social Outcomes Index (20), Brief Psychiatric Rating Scale (BPRS) (21), Time Use Survey (22), Time Use Survey (22), Health of the Nation Outcome Scale (23) and Scale to Assess Therapeutic Relationship – service user (24)] through collection of data from service users, support staff and service managers at recruitment, and 6 and 12 months after recruitment as shown in **Table 1**.

**Table 1:** Data collection summary.

Outcome measure	Assessment of	Gathered from
**Recruitment**		
Proforma	Sociodemographic details	Service user (+ case notes)
Brief Psychiatric Rating Scale (BPRS) (21)	Symptoms	Service user
Manchester Short Assessment of Quality of Life (MANSA) (16)	Quality of life	Service user
Time Use Survey (22)	Activities	Service user
Social Outcomes Index (20)	Social outcomes	Service user
Life Skills Profile (19)	Social function	Staff
Health of the Nation Outcome Scale (23)	Clinical status	Staff
Time Use Survey (22)	Activities	Staff
Clinician Alcohol and Drugs Scale (18)	Substance misuse	Staff
**6-month Follow-up**		
Time Use Survey (22)	Activities	Service user
Scale to Assess Therapeutic Relationship – service user (24)	Engagement	Service user
Time Use Survey (22)	Activities	Staff
Scale to Assess Therapeutic Relationship – clinician (24)	Engagement	Staff
**12-month Follow-up**		
Brief Psychiatric Rating Scale (21)	Symptoms	Service user
Manchester Short Assessment of Quality of Life (19)	Quality of life	Service user
Time Use Survey (22)	Activities	Service user
Social Outcomes Index (20)	Social outcomes	Service user
Client Assessment of Treatment – Supported Accommodation version (14)	Satisfaction with care	Service user
Scale to Assess Therapeutic Relationship – service user (24)	Engagement	Service user
Life Skills Profile (19)	Social function	Staff
Health of the Nation Outcome Scale (23)	Clinical status	Staff
Time Use Survey (22)	Activities	Staff
Clinician Alcohol and Drugs Scale (18)	Substance misuse	Staff
Scale to Assess Therapeutic Relationship – Clinician (24)	Engagement	Staff
Client Service Receipt Inventory (25)	Costs of care	Service user and staff and case notes
EuroQoL – 5D (26)	Cost-effectiveness	Service user

### Qualitative Evaluation

An additional qualitative component was conducted to identify themes to inform the feasibility of a larger trial. We aimed to interview 10 service user participants and 10 staff to explore their experiences of the trial, the process of randomisation, and their views on the usefulness and feasibility of a larger-scale trial. Interviews were recorded, independently transcribed, and anonymised. Text data were entered into a software package (NVivo v.10 (27)) to assist with management and coding. The interviews were analysed using thematic content analysis; a coding frame was developed by one of the researchers (RMc), with supervision from SP and GL, which was expanded and modified to include further codes as new themes and sub-themes emerged in the course of interviews and analysis.

### Data Analysis

We followed CONSORT guidelines on the analysis of feasibility trials for the presentation of our results (28). Our analysis was mainly descriptive and focused on the recruitment rate, acceptability of randomisation to participants and staff, ease of collection of data, characteristics of participants, other baseline and outcome variables, the feasibility of masking outcome assessments, loss to follow-up, and any adverse events.

### Cost-Effectiveness

Service use in the 3 months before follow-up was assessed through service user interviews and corroborated by staff and case note review, using the Client Service Receipt Inventory (25) and combined with unit costs obtained from the service manager. Service costs were derived from expenditure data (29). An instrument used to measure quality of life, the EuroQoL-5 Dimension (EQ-5D) (26), was completed with service users at 12 months follow-up for assessment of cost-effectiveness.

### Role of the Funding Source

The study was funded by the National Institute of Health Research (RP-PG-0610-10097). The funders had no role in the collection, analysis, or interpretation of data; in the writing of the manuscript; or in the decision to submit for publication. The views expressed are those of the authors and not necessarily those of the NHS, the NIHR or the Department of Health.

### Ethics Approval

The study was approved by the Liverpool Central Research Ethics Committee (ref. 15/NW/0252).

## Results

### Feasibility Metrics

**Figure 1:**
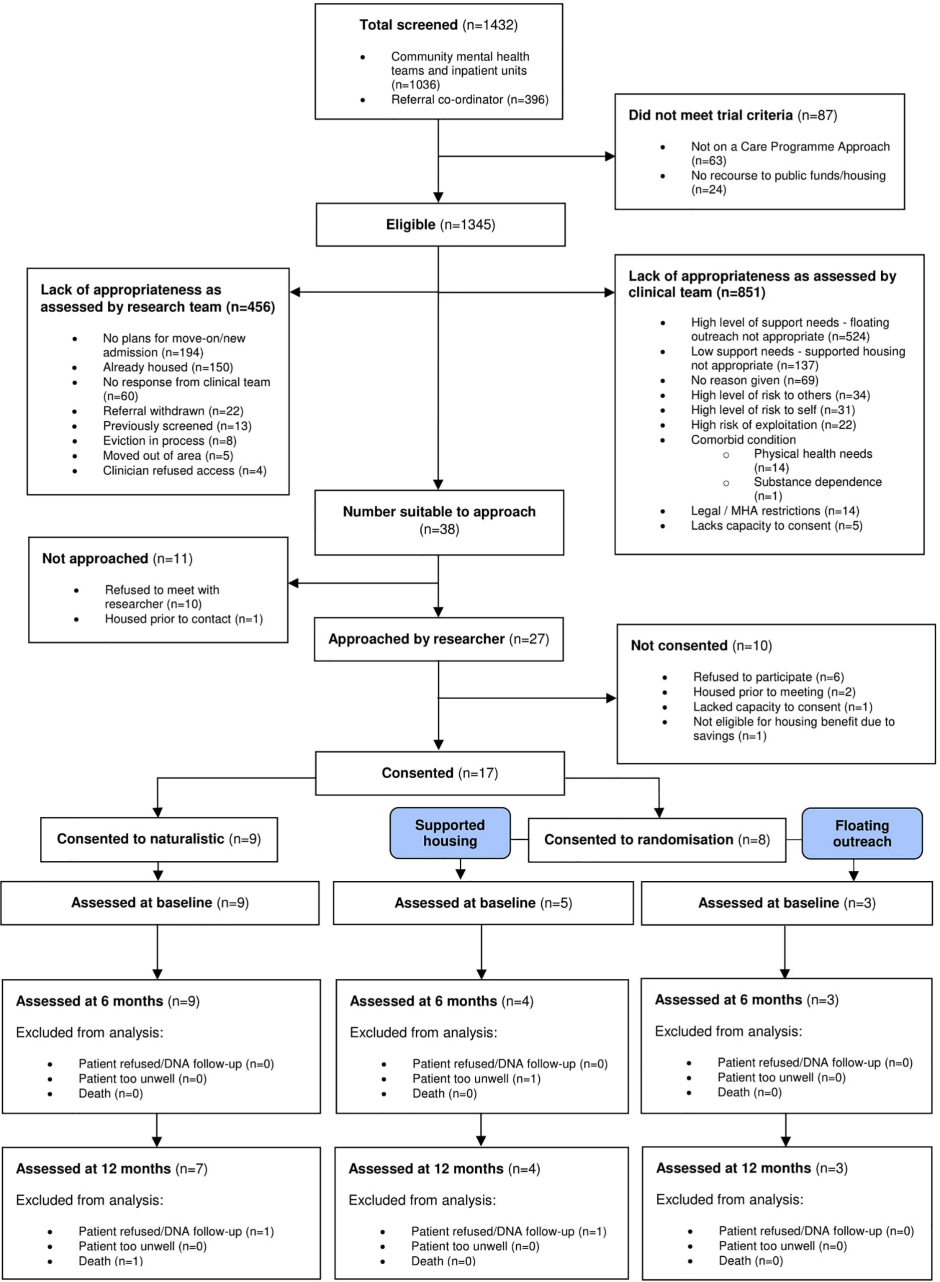
CONSORT diagram.

We screened 1,432 potential participants, of whom 87 were ineligible (not subject to the Care Programme Approach: n = 63; no recourse to public funds/housing: n = 24). Of the remaining 1,345,456 were assessed as inappropriate for participation by the researchers (no plans for move-on/new admission: n = 194; already housed: n = 150; no response from clinical team: n = 60; referral withdrawn: n = 22; previously screened: n = 13; eviction in process: n = 8; moved out of area: n = 5; clinician refused access: n = 4). A further 851 were deemed inappropriate for participation by their clinical team. The most common reasons were that the individual was felt to have a high level of support needs and was inappropriate for floating outreach (n = 524) or that they had low support needs and were inappropriate for supported housing (n = 137). In total, 17 service users consented to participate, with 8 agreeing to randomisation and 9 participating in the naturalistic arm. Participant flows are shown in **Figure 1**.

### Attrition

Of the 17 recruited participants, 3 were lost to follow-up [1 randomised (declined follow-up); 2 naturalistic group (1 died; 1 declined follow-up)].

### Time from Recruitment to Moving

Of the participants who were randomised, 3/8 (38%) moved to their allocated supported accommodation, which was supported housing in each case. This information was collected for the one randomised participant who declined follow-up via the key member of staff (they had consented to these data being collected via staff at recruitment). The median time from recruitment to moving was 4 months (IQR = 1.5–5.5). Of the five remaining randomised participants, three moved to another supported accommodation service, but not the service type they had been randomly allocated to (all three moved to a supported housing service when they were randomly allocated to move to floating outreach), one moved to their family home, and one was admitted to the hospital. Of the nine participants recruited to the naturalistic group, three moved to a supported housing service, one remained in their original supported housing service, one moved to an independent tenancy with floating outreach support, three moved to an independent tenancy without floating outreach support, and one died.

### Content of Care

The Quality Indicator for Rehabilitative Care – Supported Accommodation version (QuIRC-SA) (12) was completed with the managers of the three services that participants moved to (data not reported).

*Participant characteristics*. The mean age of participants was 38.8 years (SD = 10.1), most were male (12/17, 71%), almost half were white (8/17, 47%), and most had a diagnosis of schizophrenia (14/17, 82%). Participants’ characteristics are presented in **Table 2**.

**Table 2:** Demographic characteristics of participants at recruitment.

	Naturalistic (N = 9)	Randomised (N = 8)	Overall (N = 17)
**Age (years), mean (SD)**	38·8 (10.7)	38·9 (10.1)	38·8 (10.1)
**Male**	6 (66.7)	6 (75.0)	12 (70.6)
**Ethnicity—white**	4 (44.4)	4 (50.0)	8 (47.1)
**Diagnosis**			
Schizophrenia	8 (88.9)	6 (75.0)	14 (82.4)
Bipolar disorder	1 (11.1)	1 (12.5)	2 (11.8)
Post-traumatic stress disorder	0 (0.0)	1 (12.5)	1 (5.9)
**Length of contact with services (years), mean (SD)**	12·6 (9.6)	8·3 (6.3)	10·5 (8.3)
**Previous accommodation**			
House/flat (owner occupied)	2 (22.2)	1 (12.5)	3 (17.6)
House/flat (housing association/council)	0 (0.0)	1 (12.5)	1 (5.9)
House/flat (private rent)	2 (22.2)	2 (25.0)	4 (23.5)
Hostel/group home	0 (0.0)	4 (50.0)	4 (23.5)
Sheltered housing	3 (33.3)	0 (0.0)	3 (17.6)
Residential home	1 (11.1)	0 (0.0)	1 (5.9)
Hospital ward	1 (11.1)	0 (0.0)	1 (5.9)

*Collection of outcome data.* Data completion rates were high (100% at recruitment, 76–100% at both follow-up points). The Time Use Survey (22) and the Scale to Assess Therapeutic Relationship (24) had the lowest completion rates. The completeness of data collection is presented in **Table 3**. Due to the small numbers of participants recruited, it was not possible to conduct any quantitative outcome analyses.

**Table 3:** Completeness of data collection at each time point.

Outcome measure	Interviewee	% of participants providing data (*N* = 17)	Mean % of scale completed
**Baseline**			
Brief Psychiatric Rating Scale	Service user	17 (100%)	100%
Manchester Short Assessment of Quality of Life	Service user	17 (100%)	90%
Time Use Survey	Service user	17 (100%)	100%
Social Outcomes	Service user	17 (100%)	99%
EQ-5D	Service user	17 (100%)	100%
Client Service Receipt Inventory	Service user	17 (100%)	—
Life Skills Profile	Staff	17 (100%)	98%
Health of the Nation Outcome Scale	Staff	17 (100%)	98%
Time Use Survey	Staff	17 (100%)	100%
Clinician Alcohol and Drugs Scale	Staff	17 (100%)	100%
**6-month Follow-up**			
Time Use Survey	Service user	16 (94%)	94%
Scale to Assess Therapeutic Relationship – patient	Service user	15 (88%)	88%
Time Use Survey	Staff	14 (82%)	78%
Scale to Assess Therapeutic Relationship – clinician	Staff	17 (100%)	100%
**12-month Follow-up**			
Brief Psychiatric Rating Scale	Service user	14 (82%)	82%
Manchester Short Assessment of Quality of Life	Service user	14 (82%)	75%
Time Use Survey	Service user	14 (82%)	82%
Social Outcomes	Service user	14 (82%)	82%
Client Assessment of Treatment – Supported Accommodation version	Service user	13 (76%)	74%
Scale to Assess Therapeutic Relationship – patient	Service user	13 (76%)	76%
EQ-5D	Service user	14 (82%	100%
Client Service Receipt Inventory	Service user	14 (82%	—
Life Skills Profile	Staff	15 (88%)	88%
Health of the Nation Outcome Scale	Staff	15 (88%)	85%
Time Use Survey	Staff	11 (65%)	65%
Clinician Alcohol and Drugs Scale	Staff	15 (88%)	88%
Scale to Assess Therapeutic Relationship – clinician	Staff	14 (82%)	82%

### Masking of Researchers

Telephone administration of the MANSA (16) by a researcher who was unaware of the participant’s supported accommodation allocation was completed successfully (without revealing the allocation) for all seven randomised participants interviewed at 12 months follow-up.

### Harms/Unintended Consequences

No harms or unintended consequences occurred during the study.

### Economic Evaluation

The Client Service Receipt Inventory (CSRI) (25) and EQ-5D (26) data were collected at recruitment and 12-month follow-up. Due to the very low numbers, it was not feasible to explore any cost-effectiveness analyses.

### Qualitative Findings

We interviewed 11 service user participants (5 randomised and 6 from the naturalistic group) and 10 staff (6 care coordinators who referred participants to the study and 4 who did not). Four main themes emerged from the service user and staff interviews that helped to explain the impediments to recruitment: preference for a certain type of supported accommodation, rejection of randomisation, complexity of randomisation and value of the trial.

### Preference for a Certain Type of Supported Accommodation

Staff interviews revealed a strongly held belief that supported housing and floating outreach offered very different types of support to individuals and they therefore struggled to consider an individual as potentially suitable for either service. Thus, although there was clinical equipoise in the research literature, this was not the case for staff who usually had fixed views on the most appropriate accommodation for each patient. In particular, they stated that service users would be insufficiently supported in floating outreach and might be vulnerable to exploitation or relapse.

‘…when a decision is made to move someone into (…) an independent council flat with floating support, clinically we’ve already made the decision that you don’t think … it’s going to be a waste of resources…. Because there’s clinical reasons why you’d refer someone to a 9 to 5 [sic – supported housing] project. I’d be slightly worried about medication compliance or maybe slightly worried about safeguarding issues.’

(Staff: 2998. Referrer. Male)

Staff commonly described the two models as sequentially operating components of a ‘step-down model’, enabling staff and service users to be confident that the person could manage an independent tenancy before referral to floating outreach.

‘…the structure we’ve got does work quite well because they are in [supported accommodation provider], they stay with the staff, they are tested in the 24hr [supported housing service], they are tested in the low [floating outreach service], and then off to their own flat. It’s not a bad programme really.’

(Staff: 0020. Referrer. Male)

Whilst some service user participants had a clear preference for either floating outreach or supported housing, others appeared to see advantages and disadvantages for both types, regardless of agreement to randomisation. Service users who expressed a preference for floating outreach felt this model would permit greater autonomy.

‘I’m [forties] years of age, I’m fed up of being monitored. I’m quite able, I can cook. I can clean. I can look after myself. I can wash my clothes. I can have a bath. I can do everything on my own.’

(Service user: 5010. Naturalistic. Female)

Some consented to randomisation to increase their chance of moving to their own, permanent tenancy. For others, the preference for floating outreach permitted greater control over residential location since the process of applying for a permanent tenancy in England takes account of the individual’s preferred area. Preference was often determined by proximity to friends and family, or avoidance of areas known to have individuals who might exploit them or offer them illicit substances.

‘I like to be close to my family, you know, my daughter round, you know, my grandchildren, things like that. I thought [borough] or somewhere like that I’d like to live, if it was like that.’

(Service user: 4014. Naturalistic. Female)

‘Well I was worried that I would end up in a bad area of town … I might get involved in drugs again.’

(Service user: 5050. Naturalistic. Male)

In some cases, preference for floating outreach was influenced by family and staff. For others, previous negative experiences in supported housing persuaded them that floating outreach was preferable. Service users who preferred supported housing lacked confidence in managing without staff on-site and expressed concerns about relapse and ‘moving backwards’ if they were to move to a tenancy with floating outreach support.

‘I’m not ready for my flat yet, but everyone is saying I’m ready for it, but I’m not ready…. I just want that extra six months to make sure that I’m stable. I don’t want to get my flat and become unwell again. It costs the government so much money.’

(Service user: 2049. Randomised. Male)

Others felt that the lack of available tenancies would mean that they would wait longer for a floating outreach option than supported housing. Avoidance of isolation was also a consideration.

‘I think supported housing is better for some people … I prefer supported because you’re surrounded with people.’

(Service user: 4014. Naturalistic. Female)

Service user preferences, a lack of availability of independent tenancies leading to delays in individuals moving to floating outreach services, and a perceived lack of staff resources to facilitate service users taking part in the feasibility trial were also cited by staff as impediments to recruitment into the study.

### Rejection of Randomisation

The randomisation of accommodation was a major concern for service users and staff with the former suggesting that housing was too important to decide by chance. Staff often reflected that a (perceived) lack of equipoise between supported housing and floating outreach services made random allocation inappropriate.

‘It’s a bit … We’re talking about someone’s home here, do you know what I mean? It’s a base need. It seems like something quite serious to flip a coin about, if you know what I mean?’

(Service user: 0033. Randomised. Male)

‘It’s a question of a gradual, graduated move. So they are not really equivalent, the more I think about it, [floating outreach] or [supported housing], because there’s just more monitoring…’

(Staff: 0020. Referrer. Male)

‘So, yes I understand the randomisation process, but I would hate to think that it was to the detriment of the wellbeing of a client in a sense. There must be some clinical judgement based on where that client goes.’

(Staff: 5010. Referrer. Female)

Specifically, staff suggested that the levels of support and oversight provided by the different support types may be inappropriate to different levels of individual need. Thus, people with high needs may fail to recover, or relapse, if randomised to their own tenancy with floating outreach.

‘…he moved to a step down [from supported housing] within an organisation with floating support. Within two weeks he had a psychotic breakdown, he barricaded himself in the flat. He couldn’t cope without the staff. It was a real backward step…’

(Staff: 0020. Referrer. Male)

Service users also stated that the individual and clinician should have the final say over housing and support arrangements. Similarly, staff were concerned that randomisation negated clinical judgement in these issues and excluded the service user from valuable decision-making processes.

### Complexity of Randomisation

Despite providing informed consent for participation at recruitment, a few service users had difficulty in recalling the processes relating to randomisation during the qualitative interviews some months later. Some staff felt that the process of randomisation was too complicated for service users to understand and that this could lead to confusion or disappointment if they were allocated to a service they did not wish to move to. However, staff also struggled with understanding the trial process.

‘The first time I heard about [the trial] I thought maybe it was a platform to find a way of how our clients can get accommodation easily. That’s what I initially thought, but obviously, as you indicated, it’s not about them, it’s about basically the support they can get once they get that accommodation. Yeah. That’s what I thought.’

(Staff: 0033. Referrer. Male)

### Value of a Trial

Despite the many obstacles to recruitment we encountered, all those who participated in the qualitative interviews felt a larger trial would be worthwhile.

‘It’s helpful; you need to find out things about people who are unwell and to better things in the future to come through us who are unwell. I don’t mind helping that.’

(Service user: 2017. Naturalistic [supported housing]. Male)

## Discussion

We conducted a feasibility trial to inform whether a future large-scale randomised trial would be possible to compare the effectiveness of two commonly used models of supported accommodation that have been shown to offer similar levels of support (supported housing and floating outreach). We screened over 1,400 potential participants, but recruited only 8 who agreed to randomisation (and 9 who agreed to participate in the naturalistic group). There was a very high level of ‘gate keeping’ by staff in that many potential participants were not approached as they were deemed by their clinical team to be clinically inappropriate for the study. Of those recruited, few were lost to follow-up but few moved to their allocated accommodation and it took many weeks for the move to happen.

The outcome measures we chose were acceptable and completion rates were high. Our use of a second rater to collect follow-up data for one of our outcome measures by telephone to ensure masking proved feasible. However, the very low recruitment meant it was not possible to use our outcome data to estimate a sample size for a large-scale trial.

Our process evaluation indicated that the main obstacles to recruitment were service user preferences for a certain type of supported accommodation and a deeply ingrained belief amongst staff that individuals needed to graduate through the existing ‘step-down’ pathway, from supported housing to floating outreach, despite evidence that both have similar levels of staff support. Of note, all six participants who agreed to randomisation and subsequently moved to supported accommodation moved to supported housing, despite three being randomly allocated to move to floating outreach services. For staff, randomisation appeared to compromise their sense of professional judgement. Nevertheless, staff and service users generally felt that a large-scale trial would be valuable.

Our findings highlighted the difficulties of using a randomised trial to compare models of mental health supported accommodation. We made extensive efforts to engage potential referrers and broadened our approach to identify potential participants before the relevant clinicians had started to discuss supported accommodation options with them. However, we failed to convince staff and patients that it was ethical and safe to be recruited to the trial. Availability of supported accommodation places also influenced participation.

Although the evidence to date suggests that clinical equipoise exists between the two types of supported accommodation we included, staff had strong views based on their own experience, which clearly influenced recruitment. Patients also held their own preferences for different supported accommodation services, but these were sometimes also influenced by professionals. Although understandable, this is a major problem that needs to be overcome if we are to evaluate the effectiveness of these services. The history of medicine and medical services has shown time and again that professionals can be mistaken in their views and that clinical opinion is not a good basis on which to plan or provide services. Unfortunately, the ‘gate keeping role’ exerted by clinical staff currently means that we cannot assess robustly the most effective supported accommodation models for people with severe and complex mental health problems in the English context.

Randomised controlled trials are widely considered to be the evidence ‘gold standard’. However, alternatives are clearly needed where trials are not feasible. Well-conducted observational studies have been found to produce similar results when compared to randomised trials addressing similar research questions (30) and may therefore be appropriate in such situations. As part of the larger QuEST research programme, of which this feasibility trial comprised one component, a large, naturalistic, prospective, 30-month cohort study was carried out to assess outcomes for individuals recruited from a nationally representative sample of supported accommodation services. The findings from the cohort study will provide useful insights into the potential value of this type of study design in the field of supported accommodation research.

## Conclusion

Our feasibility trial identified a lack of acceptance amongst staff and service users of the clinical equipoise of the two models of supported accommodation being compared that resulted in insurmountable obstacles to recruitment. Our results confirmed the logistic difficulties of conducting trials in this field and help to explain the lack of randomised trials identified in systematic reviews (10, 11). Our results do not support investment in a large-scale trial in England at this time.

## Ethics Statement

The study was approved by the Liverpool Central Research Ethics Committee (ref. 15/NW/0252).

## Author contributions

HK, SP, MK, SE, PMcC, MA, SC, GL and RMa conceived and designed the study. SD, IH, JK, PMcP, CDL and RMc collected and collated the data, which were analysed by ZZ with supervision from SE. PMcC carried out the health economic analysis. All authors were involved in the interpretation of the data. HK drafted the article, which was reviewed and revised by all authors. All authors approved the final version of the manuscript and agreed their accountability in ensuring that any questions related to the accuracy or integrity of any part of the work were appropriately investigated and resolved.

## Funding

The study was funded by the National Institute of Health Research (RP-PG-0707-10093). The funders had no role in the collection, analysis or interpretation of data; in the writing of the manuscript; or in the decision to submit for publication. The views expressed are those of the authors and not necessarily those of the NHS, the NIHR or the Department of Health.

## Conflict of Interest Statement

HK, SP, MK, SE, PMcC, MA, SC, GL and GS report a grant from the National Institute of Health Research during the conduct of the study.

The remaining authors declare that the research was conducted in the absence of any commercial or financial relationships that could be construed as a potential conflict of interest.
